# Poly[bis­[μ_2_-1,3-bis­(pyridin-4-yl)urea-κ^2^ *N* ^4^:*N* ^4′^]bis­(μ_2_-5-methyl­isophthalato-κ^2^ *O* ^1^:*O* ^3^)dizinc(II)], a parallel inter­penetrated slab-like coordination polymer with {3.6^4^8}{3^2^6.7^2^8} 4,4-connected binodal topology

**DOI:** 10.1107/S2414314623006600

**Published:** 2023-08-04

**Authors:** Jason Jia, Robert L. LaDuca

**Affiliations:** a E-35 Holmes Hall, Michigan State University, Lyman Briggs College, 919 E. Shaw Lane, East Lansing, MI 48825, USA; University of Kentucky, USA

**Keywords:** crystal structure, coordination polymer, {3.6^4^8}{3^2^6.7^2^8} 4,4-connected binodal topology

## Abstract

In the title compound, [Zn_2_(C_9_H_6_O_4_)_2_(C_11_H_10_N_4_O)_2_]_*n*_, diperiodic coordination polymer slabs with {3.6^4^8}{3^2^6.7^2^8} 4,4-connected binodal topology are held into a parallel inter­penetrated triperiodic crystal structure by means of N—H⋯O hydrogen-bonding patterns.

## Abstract 2

A divalent zinc one-dimensional ribbon coordination polymer, [Zn_2_(tbuip)_2_(bpu)]_*n*_, was structurally characterized by single-crystal X-ray diffraction.

## Graphical abstract

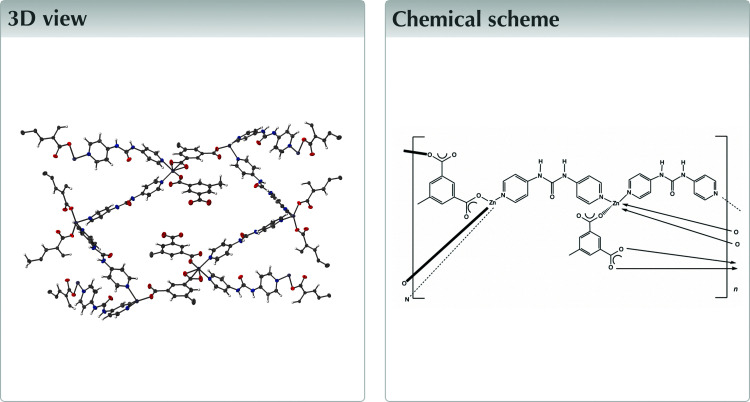

## Structure description

The title compound was isolated during an exploratory synthetic effort aiming to produce a zinc coordination polymer containing both 5-methyl­isophthalate (mip) and 4,4′-di­pyridyl­urea (dpu) ligands. Previously our group had isolated a zinc mip coordination polymer featuring 3-pyridyl­nicotinamide coligands; this phase manifested a mono-periodic ribbon structure (Kraft *et al.*, 2015 ▸).

**Figure 1:**
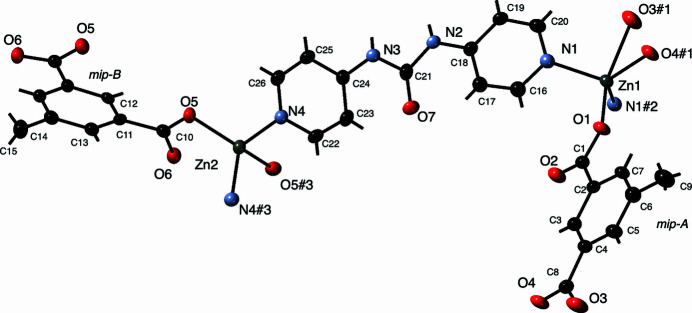
Distinct coordination environments in the title compound with full ligand sets. Displacement ellipsoids are drawn at the 50% probability level. Color code: Zn, gray; O, red; N, light blue; C, black. H atom positions are shown as sticks. Symmetry codes are as listed in Table 1 ▸.

The asymmetric unit of the title compound contains two divalent Zn atoms on special positions in space group *Pbcm*, half of one methyl­isophthalate ligand, all of whose C and O atoms are situated on a horizontal crystallographic mirror plane (mip-A), half of a second methyl­isophthalate ligand bis­ected vertically by another crystallographic mirror plane cutting through atoms C12, C14, and C15 (mip-B), and a complete dpu ligand. The Zn1 atoms are located on a crystallographic mirror plane (Wyckoff position *d*), while the Zn2 atoms are located on a crystallographic twofold rotation axis (Wyckoff position *c*). The Zn1 atoms display a five-coordinate [N_2_O_3_] environment inter­mediate between idealized trigonal–bipyramidal and square-pyramidal arrangements, as indicated by the trigonality factor τ of 0.471 (Addison & Rao, 1984 ▸). The Zn2 atoms show a *pseudo-*tetra­hedral [N_2_O_2_] environment. A depiction of the different coordination environments and full ligand set is shown in Fig. 1 ▸; numerical details are collated in Table 1 ▸.

**Figure 2:**
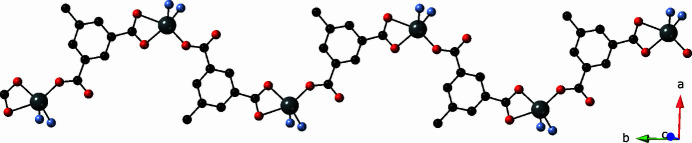
[Zn(mip)]_*n*_ coordination polymer chain in the title compound, based on Zn1 atoms and chelating/monodentate mip-A ligands.

**Figure 3:**
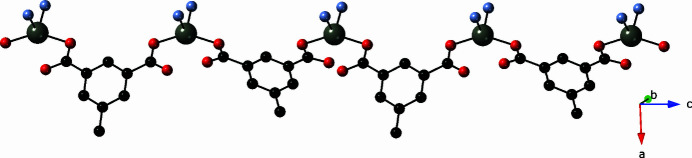
[Zn(mip)]_*n*_ coordination polymer chain in the title compound, based on Zn2 atoms and bis­(monodentate) mip-B ligands.

**Figure 4:**
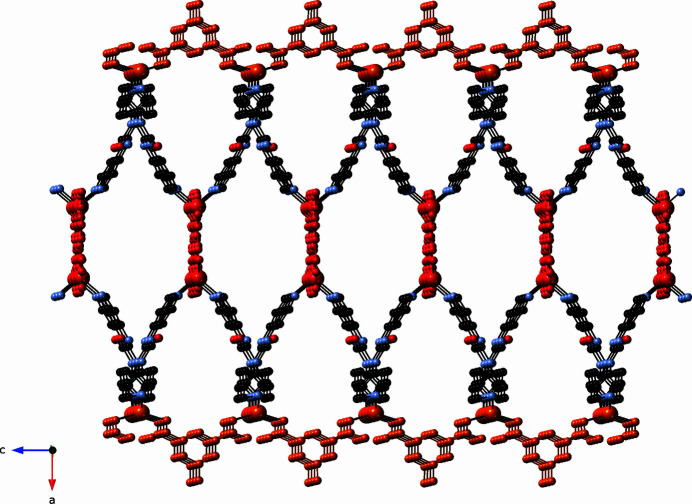
[Zn_2_(mip)_2_(bpu)_2_]_*n*_ coordination polymer slab motif in the title compound. [Zn(mip)]_*n*_ chains based on Zn1 and mip-A ligands are in the inter­ior of the slab and drawn in red. [Zn(mip)]_*n*_ chains based on Zn2 and mip-B ligands are on the exterior of the slab and drawn in orange.

**Figure 5:**
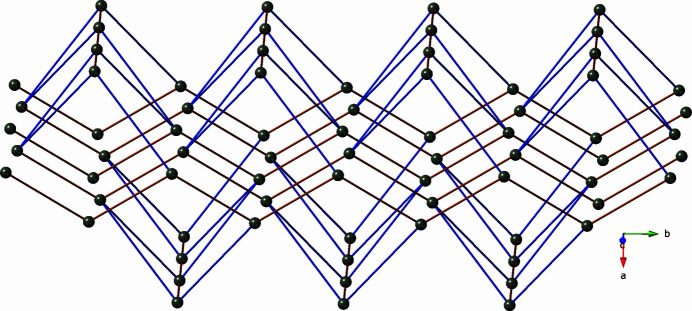
Topological perspective of a single [Zn_2_(mip)_2_(bpu)_2_]_*n*_ coordination polymer slab with {3.6^4^8}{3^2^6.7^2^8} 4,4-connected binodal topology in the title compound. The 4-connected zinc atom nodes are shown as spheres. The mip and bpu ligands are rendered as rods.

The Zn1 atoms and chelating/monodentate mip-A ligands form [Zn(mip)]_*n*_ coordination polymer chains with a Zn1⋯Zn1 distance of 10.361 (1) Å; these chain motifs are oriented along the *b* axis (Fig. 2 ▸). The Zn2 atoms and bis(monodentate) mip-B ligands form [Zn(mip)]_*n*_ coordination polymer chains with a Zn2⋯Zn2 distance of 8.746 (1) Å; these chain motifs are oriented along the *c* axis (Fig. 3 ▸). The Zn1-based Zn(mip)]_*n*_ chains and Zn2-based Zn(mip)]_*n*_ chains are oriented orthogonally to each other. In turn, these chain motifs are pillared into [Zn(mip)(bpu)]_*n*_ coordination polymer slabs by tethering bpu ligands (Fig. 4 ▸). The bpu ligands span a Zn⋯Zn distance of 13.803 (1) Å. Treating each of the Zn1 and Zn2 atoms as 4-connected nodes reveals an unprecedented {3.6^4^8}{3^2^6.7^2^8} 4,4-connected binodal topology (Fig. 5 ▸) as determined by *TOPOS* software (Blatov *et al.*, 2014 ▸).

**Figure 6:**
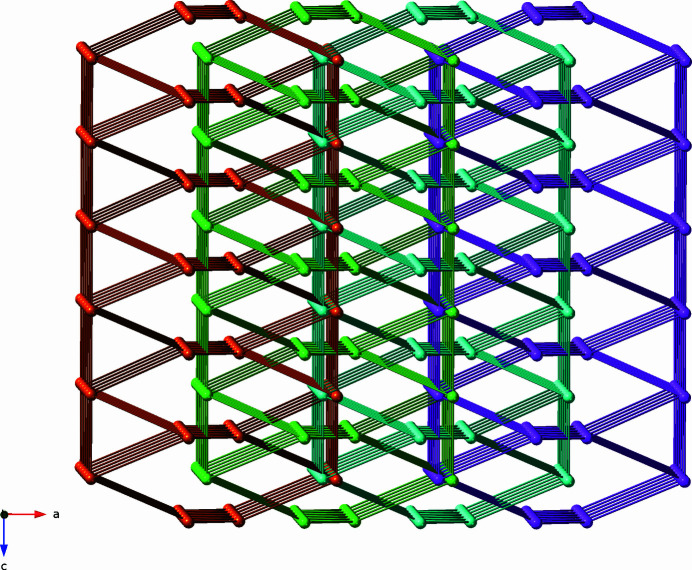
Topological perspective of the parallel inter­penetration of [Zn_2_(mip)_2_(bpu)_2_]_*n*_ coordination polymer slabs in the title compound. Each slab is depicted in a different color in order to show more clearly the entanglement between neighboring slabs.

Parallel inter­penetration of the slab motifs occurs within the title compound (Fig. 6 ▸). N—H⋯O hydrogen-bonding patterns between the central N3—H3 and N4—H4 groups of the dpu ligands and unligated mip-B carboxyl­ate O6 atoms stabilize the entangled triperiodic crystal structure. Details regarding the hydrogen bonding patterns in the title compound are listed in Table 2 ▸.

## Synthesis and crystallization

Zn(NO_3_)_2_·6H_2_O (110 mg, 0.37 mmol), 5-methyl­isophthalic acid (mipH_2_) (66 mg, 0.37 mmol), 4,4′-di­pyridyl­urea (dpu) (79 mg, 0.37 mmol), and 0.75 ml of a 1.0 *M* NaOH solution were placed into 10 ml of distilled water in a Teflon-lined acid digestion bomb. The bomb was sealed and heated in an oven at 393 K for 48 h, and then cooled slowly to 273 K. Colorless crystals of the title complex were obtained in 59% yield.

## Refinement

Crystal data, data collection and structure refinement details are summarized in Table 3 ▸.

## Supplementary Material

Crystal structure: contains datablock(s) I, 1R. DOI: 10.1107/S2414314623006600/pk4042sup1.cif

Structure factors: contains datablock(s) I. DOI: 10.1107/S2414314623006600/pk4042Isup2.hkl

CCDC reference: 1959994

Additional supporting information:  crystallographic information; 3D view; checkCIF report

## Figures and Tables

**Table 1:** Selected geometric parameters (Å, °)

Zn1—O1	1.952 (3)	Zn2—O5^iii^	2.004 (2)
Zn1—O3^i^	2.272 (3)	Zn2—O5	2.004 (2)
Zn1—O4^i^	2.060 (3)	Zn2—N4^iii^	2.045 (3)
Zn1—N1	2.077 (3)	Zn2—N4	2.045 (3)
Zn1—N1^ii^	2.077 (3)		
			
O1—Zn1—O3^i^	154.66 (12)	N1—Zn1—O3^i^	91.12 (9)
O1—Zn1—O4^i^	94.69 (13)	N1^ii^—Zn1—N1	95.14 (14)
O1—Zn1—N1^ii^	105.72 (9)	O5—Zn2—O5^iii^	145.53 (12)
O1—Zn1—N1	105.72 (9)	O5—Zn2—N4^iii^	102.20 (9)
O4^i^—Zn1—O3^i^	59.97 (12)	O5^iii^—Zn2—N4^iii^	99.41 (9)
O4^i^—Zn1—N1	126.42 (8)	O5—Zn2—N4	99.41 (9)
O4^i^—Zn1—N1^ii^	126.42 (8)	O5^iii^—Zn2—N4	102.20 (9)
N1^ii^—Zn1—O3^i^	91.13 (9)	N4^iii^—Zn2—N4	101.55 (14)

Symmetry codes: (i) 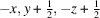 ; (ii) 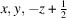 ; (iii) 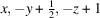 .

**Table 2:** Hydrogen-bond geometry (Å, °)

*D*—H⋯*A*	*D*—H	H⋯*A*	*D*⋯*A*	*D*—H⋯*A*
N2—H2⋯O6^iv^	0.88	2.12	2.919 (3)	151
N3—H3⋯O6^iv^	0.88	1.91	2.751 (3)	159

Symmetry code: (iv) 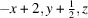 .

**Table 3:** Experimental details

Crystal data	
Chemical formula	[Zn_2_(C_9_H_6_O_4_)_2_(C_11_H_10_N_4_O)_2_]
*M*_r_	915.47
Crystal system, space group	Orthorhombic, *P* *b* *c* *m*
Temperature (K)	173
*a*, *b*, *c* (Å)	12.0755 (11), 17.7239 (16), 17.4919 (16)
*V* (Å^3^)	3743.7 (6)
*Z*	4
Radiation type	Mo *K*α
μ (mm^−1^)	1.36
Crystal size (mm)	0.29 × 0.16 × 0.11

Data collection	
Diffractometer	Bruker APEXII CCD
Absorption correction	Multi-scan (*SADABS*; Krause *et al.*, 2015 ▸)
*T*_min_, *T*_max_	0.654, 0.745
No. of measured, independent and observed [*I* > 2σ(*I*)] reflections	29050, 3546, 2731
*R*_int_	0.068
(sin θ/λ)_max_ (Å^−1^)	0.602

Refinement	
*R*[*F* ^2^ > 2σ(*F* ^2^)], *wR*(*F* ^2^), *S*	0.038, 0.096, 1.04
No. of reflections	3546
No. of parameters	299
H-atom treatment	H-atom parameters constrained
Δρ_max_, Δρ_min_ (e Å^−3^)	0.88, −0.34

Computer programs: *COSMO* (Bruker, 2009 ▸), *SAINT* (Bruker, 2014 ▸), *SHELXT* (Sheldrick, 2015*a* ▸), *SHELXL* (Sheldrick, 2015*b* ▸), *Crystal Maker X* (Palmer, 2020 ▸), and *OLEX2* (Dolomanov *et al.*, (2009 ▸).

## full crystallographic data

### Crystal data

**Table:**

[Zn_2_(C_9_H_6_O_4_)_2_(C_11_H_10_N_4_O)_2_]	*D*_x_ = 1.624 Mg m^−^^3^
*M**_r_* = 915.47	Mo *K*α radiation, λ = 0.71073 Å
Orthorhombic, *P**b**c**m*	Cell parameters from 8405 reflections
*a* = 12.0755 (11) Å	θ = 2.3–25.2°
*b* = 17.7239 (16) Å	µ = 1.36 mm^−^^1^
*c* = 17.4919 (16) Å	*T* = 173 K
*V* = 3743.7 (6) Å^3^	Block, colourless
*Z* = 4	0.29 × 0.16 × 0.11 mm
*F*(000) = 1872	

### Data collection

**Table:**

Bruker APEXII CCD diffractometer	3546 independent reflections
Radiation source: sealed tube	2731 reflections with *I* > 2σ(*I*)
Graphite monochromator	*R*_int_ = 0.068
Detector resolution: 8.36 pixels mm^-1^	θ_max_ = 25.3°, θ_min_ = 1.7°
ω scans	*h* = −14→14
Absorption correction: multi-scan (SADABS; Krause *et al.*, 2015)	*k* = −20→21
*T*_min_ = 0.654, *T*_max_ = 0.745	*l* = −21→21
29050 measured reflections	

### Refinement

**Table:**

Refinement on *F*^2^	Primary atom site location: dual
Least-squares matrix: full	Hydrogen site location: inferred from neighbouring sites
*R*[*F*^2^ > 2σ(*F*^2^)] = 0.038	H-atom parameters constrained
*wR*(*F*^2^) = 0.096	*w* = 1/[σ^2^(*F*_o_^2^) + (0.0396*P*)^2^ + 4.9027*P*] where *P* = (*F*_o_^2^ + 2*F*_c_^2^)/3
*S* = 1.04	(Δ/σ)_max_ = 0.001
3546 reflections	Δρ_max_ = 0.88 e Å^−^^3^
299 parameters	Δρ_min_ = −0.34 e Å^−^^3^
0 restraints	

### Special details

**Table:**

Experimental. Data were collected using a BRUKER CCD (charge coupled device) based diffractometer equipped with an Oxford low-temperature apparatus operating at 173 K. A suitable crystal was chosen and mounted on a nylon loop using Paratone oil. Data were measured using omega scans of 0.5° per frame for 30 s. The total number of images were based on results from the program COSMO where redundancy was expected to be 4 and completeness to 0.83Å to 100%. Cell parameters were retrieved using APEX II software and refined using SAINT on all observed reflections.Data reduction was performed using the SAINT software which corrects for Lp. Scaling and absorption corrections were applied using SADABS multi-scan technique, supplied by George Sheldrick. The structure was solved by the direct method using the SHELXT program and refined by least squares method on F2, SHELXL, incorporated in OLEX2.
Geometry. All esds (except the esd in the dihedral angle between two l.s. planes) are estimated using the full covariance matrix. The cell esds are taken into account individually in the estimation of esds in distances, angles and torsion angles; correlations between esds in cell parameters are only used when they are defined by crystal symmetry. An approximate (isotropic) treatment of cell esds is used for estimating esds involving l.s. planes.
Refinement. The structure was refined by Least Squares using version 2018/3 of *SHELXL* (Sheldrick, 2015b) incorporated in *OLEX2* (Dolomanov *et al.*, 2009). All non-hydrogen atoms were refined anisotropically. Hydrogen atom positions were calculated geometrically and refined using the riding model, except for the Hydrogen atom on the nitrogen atom which was found by difference Fourier methods and refined isotropically.

### Fractional atomic coordinates and isotropic or equivalent isotropic displacement parameters (Å^2^)

**Table:**

	*x*	*y*	*z*	*U*_iso_*/*U*_eq_	Occ. (<1)
Zn1	0.22230 (4)	0.72239 (3)	0.2500	0.02069 (15)	
Zn2	1.05581 (4)	0.2500	0.5000	0.02236 (15)	
O1	0.1090 (2)	0.64386 (17)	0.2500	0.0286 (7)	
O2	0.1735 (3)	0.52565 (17)	0.2500	0.0319 (8)	
O3	−0.2841 (3)	0.34350 (18)	0.2500	0.0424 (9)	
O4	−0.1108 (3)	0.31038 (17)	0.2500	0.0338 (8)	
O5	1.10499 (17)	0.24973 (12)	0.60942 (12)	0.0261 (5)	
O6	1.21086 (18)	0.17083 (12)	0.54534 (11)	0.0282 (5)	
O7	0.59432 (19)	0.48433 (13)	0.42346 (14)	0.0378 (6)	
N1	0.3308 (2)	0.69427 (14)	0.33766 (15)	0.0245 (6)	
N2	0.6071 (2)	0.60954 (15)	0.45472 (15)	0.0280 (6)	
H2	0.6473	0.6425	0.4802	0.034*	
N3	0.7414 (2)	0.52788 (15)	0.49375 (15)	0.0273 (6)	
H3	0.7647	0.5673	0.5197	0.033*	
N4	0.9487 (2)	0.33934 (14)	0.50316 (14)	0.0242 (6)	
C1	0.0980 (4)	0.5716 (2)	0.2500	0.0226 (10)	
C2	−0.0202 (3)	0.5443 (2)	0.2500	0.0205 (9)	
C3	−0.0429 (4)	0.4671 (2)	0.2500	0.0232 (10)	
H3A	0.0159	0.4315	0.2500	0.028*	
C4	−0.1522 (4)	0.4429 (2)	0.2500	0.0236 (10)	
C5	−0.2375 (4)	0.4956 (3)	0.2500	0.0277 (10)	
H5	−0.3121	0.4785	0.2500	0.033*	
C6	−0.2167 (4)	0.5721 (3)	0.2500	0.0283 (11)	
C7	−0.1065 (3)	0.5954 (2)	0.2500	0.0232 (10)	
H7	−0.0903	0.6478	0.2500	0.028*	
C8	−0.1840 (4)	0.3612 (3)	0.2500	0.0246 (10)	
C9	−0.3097 (4)	0.6291 (3)	0.2500	0.0403 (13)	
H9A	−0.3105	0.6560	0.2989	0.061*	0.5
H9B	−0.3805	0.6030	0.2428	0.061*	0.5
H9C	−0.2984	0.6652	0.2083	0.061*	0.5
C10	1.1788 (2)	0.19887 (16)	0.60787 (17)	0.0223 (7)	
C11	1.2281 (2)	0.17337 (16)	0.68140 (16)	0.0190 (6)	
C12	1.1792 (3)	0.1945 (2)	0.7500	0.0191 (9)	
H12	1.1128	0.2232	0.7500	0.023*	
C13	1.3233 (2)	0.12961 (16)	0.68196 (17)	0.0217 (7)	
H13	1.3555	0.1146	0.6348	0.026*	
C14	1.3722 (3)	0.1073 (2)	0.7500	0.0230 (10)	
C15	1.4743 (4)	0.0582 (3)	0.7500	0.0322 (11)	
H15A	1.4537	0.0064	0.7365	0.048*	0.5
H15B	1.5080	0.0587	0.8010	0.048*	0.5
H15C	1.5276	0.0775	0.7125	0.048*	0.5
C16	0.3396 (3)	0.62065 (18)	0.35476 (19)	0.0283 (7)	
H16	0.2819	0.5879	0.3385	0.034*	
C17	0.4266 (3)	0.58995 (19)	0.39417 (18)	0.0292 (8)	
H17	0.4284	0.5374	0.4050	0.035*	
C18	0.5121 (3)	0.63642 (18)	0.41805 (17)	0.0241 (7)	
C19	0.5028 (3)	0.71332 (18)	0.40297 (19)	0.0285 (7)	
H19	0.5585	0.7473	0.4199	0.034*	
C20	0.4126 (3)	0.73950 (18)	0.36345 (19)	0.0293 (8)	
H20	0.4074	0.7921	0.3537	0.035*	
C21	0.6433 (3)	0.53544 (19)	0.45420 (18)	0.0287 (8)	
C22	0.8676 (3)	0.34385 (19)	0.45077 (19)	0.0342 (8)	
H22	0.8600	0.3036	0.4153	0.041*	
C23	0.7952 (3)	0.40320 (19)	0.44552 (19)	0.0323 (8)	
H23	0.7382	0.4034	0.4081	0.039*	
C24	0.8071 (3)	0.46316 (17)	0.49621 (18)	0.0241 (7)	
C25	0.8906 (3)	0.45927 (18)	0.55071 (18)	0.0263 (7)	
H25	0.9008	0.4992	0.5863	0.032*	
C26	0.9581 (3)	0.39689 (18)	0.55249 (18)	0.0266 (7)	
H26	1.0142	0.3944	0.5905	0.032*	

### Atomic displacement parameters (Å^2^)

**Table:**

	*U*^11^	*U*^22^	*U*^33^	*U*^12^	*U*^13^	*U*^23^
Zn1	0.0175 (3)	0.0172 (3)	0.0274 (3)	0.0009 (2)	0.000	0.000
Zn2	0.0250 (3)	0.0248 (3)	0.0173 (3)	0.000	0.000	0.0021 (2)
O1	0.0233 (16)	0.0167 (17)	0.046 (2)	−0.0039 (13)	0.000	0.000
O2	0.0214 (17)	0.0210 (17)	0.053 (2)	0.0003 (14)	0.000	0.000
O3	0.031 (2)	0.0230 (19)	0.074 (3)	−0.0118 (15)	0.000	0.000
O4	0.0318 (19)	0.0166 (17)	0.053 (2)	−0.0057 (14)	0.000	0.000
O5	0.0301 (12)	0.0267 (12)	0.0216 (11)	0.0084 (10)	−0.0043 (9)	0.0007 (9)
O6	0.0387 (14)	0.0292 (13)	0.0167 (11)	0.0069 (10)	0.0021 (10)	−0.0012 (9)
O7	0.0370 (14)	0.0308 (14)	0.0455 (15)	0.0031 (11)	−0.0199 (12)	−0.0093 (12)
N1	0.0207 (14)	0.0236 (15)	0.0292 (14)	0.0009 (11)	−0.0001 (11)	−0.0004 (12)
N2	0.0271 (15)	0.0243 (15)	0.0326 (16)	0.0021 (12)	−0.0129 (12)	−0.0045 (12)
N3	0.0299 (15)	0.0224 (14)	0.0295 (15)	0.0024 (11)	−0.0088 (12)	−0.0028 (12)
N4	0.0263 (14)	0.0250 (15)	0.0213 (14)	0.0024 (11)	−0.0020 (11)	−0.0004 (11)
C1	0.027 (2)	0.021 (2)	0.020 (2)	−0.003 (2)	0.000	0.000
C2	0.023 (2)	0.021 (2)	0.018 (2)	−0.0049 (18)	0.000	0.000
C3	0.025 (2)	0.023 (2)	0.022 (2)	−0.0022 (19)	0.000	0.000
C4	0.029 (2)	0.020 (2)	0.022 (2)	−0.0045 (19)	0.000	0.000
C5	0.023 (2)	0.023 (3)	0.037 (3)	−0.0068 (19)	0.000	0.000
C6	0.021 (2)	0.028 (3)	0.036 (3)	0.001 (2)	0.000	0.000
C7	0.023 (2)	0.017 (2)	0.029 (2)	−0.0037 (18)	0.000	0.000
C8	0.028 (2)	0.026 (3)	0.021 (2)	−0.005 (2)	0.000	0.000
C9	0.023 (3)	0.031 (3)	0.067 (4)	0.001 (2)	0.000	0.000
C10	0.0242 (16)	0.0190 (16)	0.0237 (17)	−0.0008 (13)	0.0014 (13)	0.0003 (13)
C11	0.0194 (15)	0.0186 (16)	0.0190 (15)	−0.0021 (12)	−0.0011 (12)	−0.0024 (12)
C12	0.018 (2)	0.013 (2)	0.026 (2)	−0.0001 (17)	0.000	0.000
C13	0.0239 (16)	0.0192 (16)	0.0221 (16)	0.0002 (13)	0.0028 (13)	−0.0007 (13)
C14	0.018 (2)	0.021 (2)	0.030 (2)	0.0011 (18)	0.000	0.000
C15	0.031 (3)	0.037 (3)	0.028 (3)	0.014 (2)	0.000	0.000
C16	0.0245 (17)	0.0278 (19)	0.0327 (18)	−0.0048 (14)	−0.0017 (14)	0.0039 (15)
C17	0.0270 (18)	0.0270 (18)	0.0335 (19)	−0.0026 (14)	−0.0053 (15)	0.0034 (15)
C18	0.0243 (16)	0.0272 (18)	0.0206 (15)	0.0014 (14)	−0.0014 (13)	−0.0022 (13)
C19	0.0250 (17)	0.0260 (18)	0.0344 (19)	0.0002 (14)	−0.0105 (15)	−0.0062 (15)
C20	0.0290 (18)	0.0217 (18)	0.037 (2)	0.0013 (14)	−0.0022 (15)	−0.0044 (14)
C21	0.0314 (18)	0.031 (2)	0.0240 (17)	0.0037 (15)	−0.0047 (15)	0.0031 (15)
C22	0.045 (2)	0.030 (2)	0.0277 (18)	0.0054 (16)	−0.0134 (16)	−0.0094 (15)
C23	0.0356 (19)	0.035 (2)	0.0265 (18)	0.0082 (15)	−0.0113 (15)	−0.0035 (15)
C24	0.0233 (16)	0.0253 (17)	0.0238 (16)	−0.0002 (13)	−0.0016 (13)	0.0028 (14)
C25	0.0273 (17)	0.0286 (19)	0.0230 (17)	−0.0019 (14)	−0.0041 (13)	−0.0037 (14)
C26	0.0259 (17)	0.0315 (19)	0.0224 (16)	0.0005 (14)	−0.0044 (13)	−0.0004 (14)

### Geometric parameters (Å, º)

**Table:**

Zn1—O1	1.952 (3)	C5—C6	1.379 (6)
Zn1—O3^i^	2.272 (3)	C6—C7	1.393 (6)
Zn1—O4^i^	2.060 (3)	C6—C9	1.510 (6)
Zn1—N1	2.077 (3)	C7—H7	0.9500
Zn1—N1^ii^	2.077 (3)	C8—Zn1^iv^	2.503 (5)
Zn2—O5^iii^	2.004 (2)	C9—H9A	0.9800
Zn2—O5	2.004 (2)	C9—H9B	0.9800
Zn2—N4^iii^	2.045 (3)	C9—H9C	0.9800
Zn2—N4	2.045 (3)	C10—C11	1.488 (4)
O1—C1	1.287 (5)	C11—C12	1.388 (4)
O2—C1	1.223 (5)	C11—C13	1.387 (4)
O3—Zn1^iv^	2.272 (3)	C12—C11^v^	1.389 (4)
O3—C8	1.248 (5)	C12—H12	0.9500
O4—Zn1^iv^	2.060 (3)	C13—H13	0.9500
O4—C8	1.262 (5)	C13—C14	1.386 (4)
O5—C10	1.268 (4)	C14—C13^v^	1.386 (4)
O6—C10	1.262 (4)	C14—C15	1.510 (6)
O7—C21	1.208 (4)	C15—H15A	0.9800
N1—C16	1.343 (4)	C15—H15B	0.9800
N1—C20	1.350 (4)	C15—H15C	0.9800
N2—H2	0.8800	C16—H16	0.9500
N2—C18	1.399 (4)	C16—C17	1.370 (4)
N2—C21	1.384 (4)	C17—H17	0.9500
N3—H3	0.8800	C17—C18	1.385 (4)
N3—C21	1.378 (4)	C18—C19	1.393 (4)
N3—C24	1.395 (4)	C19—H19	0.9500
N4—C22	1.343 (4)	C19—C20	1.371 (4)
N4—C26	1.341 (4)	C20—H20	0.9500
C1—C2	1.507 (6)	C22—H22	0.9500
C2—C3	1.395 (6)	C22—C23	1.371 (5)
C2—C7	1.380 (6)	C23—H23	0.9500
C3—H3A	0.9500	C23—C24	1.391 (4)
C3—C4	1.389 (6)	C24—C25	1.389 (4)
C4—C5	1.391 (6)	C25—H25	0.9500
C4—C8	1.497 (6)	C25—C26	1.374 (4)
C5—H5	0.9500	C26—H26	0.9500
			
O1—Zn1—O3^i^	154.66 (12)	C6—C9—H9C	109.5
O1—Zn1—O4^i^	94.69 (13)	H9A—C9—H9B	109.5
O1—Zn1—N1^ii^	105.72 (9)	H9A—C9—H9C	109.5
O1—Zn1—N1	105.72 (9)	H9B—C9—H9C	109.5
O4^i^—Zn1—O3^i^	59.97 (12)	O5—C10—Zn2	50.06 (14)
O4^i^—Zn1—N1	126.42 (8)	O5—C10—C11	118.6 (3)
O4^i^—Zn1—N1^ii^	126.42 (8)	O6—C10—Zn2	71.31 (16)
N1^ii^—Zn1—O3^i^	91.13 (9)	O6—C10—O5	120.9 (3)
N1—Zn1—O3^i^	91.12 (9)	O6—C10—C11	120.5 (3)
N1^ii^—Zn1—N1	95.14 (14)	C11—C10—Zn2	167.0 (2)
O5—Zn2—O5^iii^	145.53 (12)	C12—C11—C10	119.7 (3)
O5—Zn2—N4^iii^	102.20 (9)	C13—C11—C10	120.5 (3)
O5^iii^—Zn2—N4^iii^	99.41 (9)	C13—C11—C12	119.8 (3)
O5—Zn2—N4	99.41 (9)	C11—C12—C11^v^	119.6 (4)
O5^iii^—Zn2—N4	102.20 (9)	C11^v^—C12—H12	120.2
N4^iii^—Zn2—N4	101.55 (14)	C11—C12—H12	120.2
C1—O1—Zn1	141.4 (3)	C11—C13—H13	119.4
C8—O3—Zn1^iv^	85.4 (3)	C14—C13—C11	121.3 (3)
C8—O4—Zn1^iv^	94.7 (3)	C14—C13—H13	119.4
C10—O5—Zn2	100.92 (18)	C13^v^—C14—C13	118.3 (4)
C10—O6—Zn2	79.75 (17)	C13^v^—C14—C15	120.85 (19)
C16—N1—Zn1	116.6 (2)	C13—C14—C15	120.85 (19)
C16—N1—C20	116.4 (3)	C14—C15—H15A	109.5
C20—N1—Zn1	124.5 (2)	C14—C15—H15B	109.5
C18—N2—H2	117.3	C14—C15—H15C	109.5
C21—N2—H2	117.3	H15A—C15—H15B	109.5
C21—N2—C18	125.4 (3)	H15A—C15—H15C	109.5
C21—N3—H3	117.1	H15B—C15—H15C	109.5
C21—N3—C24	125.7 (3)	N1—C16—H16	118.0
C24—N3—H3	117.1	N1—C16—C17	124.0 (3)
C22—N4—Zn2	119.2 (2)	C17—C16—H16	118.0
C26—N4—Zn2	123.6 (2)	C16—C17—H17	120.4
C26—N4—C22	117.1 (3)	C16—C17—C18	119.2 (3)
O1—C1—C2	114.7 (4)	C18—C17—H17	120.4
O2—C1—O1	125.8 (4)	C17—C18—N2	123.2 (3)
O2—C1—C2	119.5 (4)	C17—C18—C19	117.7 (3)
C3—C2—C1	120.0 (4)	C19—C18—N2	119.1 (3)
C7—C2—C1	120.3 (4)	C18—C19—H19	120.3
C7—C2—C3	119.7 (4)	C20—C19—C18	119.4 (3)
C2—C3—H3A	120.3	C20—C19—H19	120.3
C4—C3—C2	119.3 (4)	N1—C20—C19	123.3 (3)
C4—C3—H3A	120.3	N1—C20—H20	118.3
C3—C4—C5	119.7 (4)	C19—C20—H20	118.3
C3—C4—C8	122.9 (4)	O7—C21—N2	124.0 (3)
C5—C4—C8	117.4 (4)	O7—C21—N3	124.9 (3)
C4—C5—H5	119.1	N3—C21—N2	111.1 (3)
C6—C5—C4	121.7 (4)	N4—C22—H22	118.1
C6—C5—H5	119.1	N4—C22—C23	123.8 (3)
C5—C6—C7	117.7 (4)	C23—C22—H22	118.1
C5—C6—C9	121.5 (4)	C22—C23—H23	120.7
C7—C6—C9	120.8 (4)	C22—C23—C24	118.5 (3)
C2—C7—C6	121.8 (4)	C24—C23—H23	120.7
C2—C7—H7	119.1	C23—C24—N3	123.4 (3)
C6—C7—H7	119.1	C25—C24—N3	118.3 (3)
O3—C8—Zn1^iv^	64.8 (2)	C25—C24—C23	118.3 (3)
O3—C8—O4	119.9 (4)	C24—C25—H25	120.5
O3—C8—C4	119.4 (4)	C26—C25—C24	119.1 (3)
O4—C8—Zn1^iv^	55.1 (2)	C26—C25—H25	120.5
O4—C8—C4	120.7 (4)	N4—C26—C25	123.2 (3)
C4—C8—Zn1^iv^	175.8 (3)	N4—C26—H26	118.4
C6—C9—H9A	109.5	C25—C26—H26	118.4
C6—C9—H9B	109.5		
			
Zn1—O1—C1—O2	0.0	C3—C4—C8—O4	0.0
Zn1—O1—C1—C2	180.0	C4—C5—C6—C7	0.0
Zn1^iv^—O3—C8—O4	0.0	C4—C5—C6—C9	180.0
Zn1^iv^—O3—C8—C4	180.0	C5—C4—C8—O3	0.0
Zn1^iv^—O4—C8—O3	0.0	C5—C4—C8—O4	180.0
Zn1^iv^—O4—C8—C4	180.0	C5—C6—C7—C2	0.0
Zn1—N1—C16—C17	160.9 (3)	C7—C2—C3—C4	0.0
Zn1—N1—C20—C19	−159.1 (3)	C8—C4—C5—C6	180.0
Zn2—O5—C10—O6	8.4 (3)	C9—C6—C7—C2	180.0
Zn2—O5—C10—C11	−172.3 (2)	C10—C11—C12—C11^v^	−177.2 (2)
Zn2—O6—C10—O5	−6.8 (3)	C10—C11—C13—C14	177.9 (3)
Zn2—O6—C10—C11	174.0 (3)	C11—C13—C14—C13^v^	0.3 (6)
Zn2—N4—C22—C23	−176.6 (3)	C11—C13—C14—C15	178.6 (4)
Zn2—N4—C26—C25	175.2 (2)	C12—C11—C13—C14	−1.1 (5)
Zn2—C10—C11—C12	−15.1 (11)	C13—C11—C12—C11^v^	1.9 (6)
Zn2—C10—C11—C13	165.9 (8)	C16—N1—C20—C19	2.1 (5)
O1—C1—C2—C3	180.0	C16—C17—C18—N2	−176.2 (3)
O1—C1—C2—C7	0.0	C16—C17—C18—C19	2.3 (5)
O2—C1—C2—C3	0.0	C17—C18—C19—C20	−2.1 (5)
O2—C1—C2—C7	180.0	C18—N2—C21—O7	−2.4 (5)
O5—C10—C11—C12	12.0 (5)	C18—N2—C21—N3	178.2 (3)
O5—C10—C11—C13	−167.0 (3)	C18—C19—C20—N1	−0.2 (5)
O6—C10—C11—C12	−168.7 (3)	C20—N1—C16—C17	−1.8 (5)
O6—C10—C11—C13	12.3 (4)	C21—N2—C18—C17	18.2 (5)
N1—C16—C17—C18	−0.4 (5)	C21—N2—C18—C19	−160.4 (3)
N2—C18—C19—C20	176.6 (3)	C21—N3—C24—C23	15.0 (5)
N3—C24—C25—C26	−178.1 (3)	C21—N3—C24—C25	−166.9 (3)
N4—C22—C23—C24	1.3 (6)	C22—N4—C26—C25	−1.0 (5)
C1—C2—C3—C4	180.0	C22—C23—C24—N3	176.9 (3)
C1—C2—C7—C6	180.0	C22—C23—C24—C25	−1.2 (5)
C2—C3—C4—C5	0.0	C23—C24—C25—C26	0.1 (5)
C2—C3—C4—C8	180.0	C24—N3—C21—O7	9.0 (5)
C3—C2—C7—C6	0.0	C24—N3—C21—N2	−171.5 (3)
C3—C4—C5—C6	0.0	C24—C25—C26—N4	1.0 (5)
C3—C4—C8—O3	180.0	C26—N4—C22—C23	−0.2 (5)

Symmetry codes: (i) −*x*, *y*+1/2, −*z*+1/2; (ii) *x*, *y*, −*z*+1/2; (iii) *x*, −*y*+1/2, −*z*+1; (iv) −*x*, *y*−1/2, −*z*+1/2; (v) *x*, *y*, −*z*+3/2.

### Hydrogen-bond geometry (Å, º)

**Table:**

*D*—H···*A*	*D*—H	H···*A*	*D*···*A*	*D*—H···*A*
N2—H2···O6^vi^	0.88	2.12	2.919 (3)	151
N3—H3···O6^vi^	0.88	1.91	2.751 (3)	159
C5—H5···O3	0.95	2.42	2.754 (5)	101
C7—H7···O1	0.95	2.41	2.741 (5)	100
C17—H17···O7	0.95	2.24	2.805 (4)	117
C23—H23···O7	0.95	2.27	2.846 (4)	118

Symmetry code: (vi) −*x*+2, *y*+1/2, *z*.
